# Uptake and Accumulation of Cobalt Is Mediated by OsNramp5 in Rice

**DOI:** 10.1111/pce.15130

**Published:** 2024-09-02

**Authors:** Hengliang Huang, Naoki Yamaji, Sheng Huang, Jian Feng Ma

**Affiliations:** ^1^ Institute of Plant Science and Resources Okayama University Kurashiki Japan

**Keywords:** cobalt, rice, safety, transporter, uptake

## Abstract

Cobalt (Co) contamination in soils potentially affects human health through the food chain. Although rice (*Oryza sativa*) as a staple food is a major dietary source of human Co intake, it is poorly understood how Co is taken up by the roots and accumulated in rice grain. In this study, we physiologically characterized Co accumulation and identified the transporter for Co^2+^ uptake in rice. A dose‐dependent experiment showed that Co mainly accumulated in rice roots. Further analysis with LA‐ICP‐MS showed Co deposited in most tissue of the roots, including exodermis, endodermis and stele region. Co accumulation analysis using mutants defective in divalent cation uptake showed that Co^2+^ uptake in rice is mediated by the Mn^2+^/Cd^2+^/Pb^2+^ transporter OsNramp5, rather than OsIRT1 for Fe^2+^ and OsZIP9 for Zn^2+^. Knockout of *OsNramp5* enhanced tolerance to Co toxicity. Heterologous expression of *OsNramp5* showed transport activity for Co^2+^ in *Saccharomyces cerevisiae*. Co^2+^ uptake was inhibited by either Mn^2+^ or Cd^2+^ supply. At the reproductive stage, the Co concentration in the straw and grains of the *OsNramp5* knockout lines was decreased by 41%–48% and 28%–36%, respectively, compared with that of the wild‐type rice. The expression level of *OsNramp5* in the roots was not affected by Co^2+^. Taken together, our results indicate that OsNramp5 is a major transporter for Co^2+^ uptake in rice, which ultimately mediates Co accumulation in the grains.

## Summary statement

OsNramp5 is a major transporter responsible for cobalt uptake and its defect reduces cobalt accumulation in the shoot and grain of rice.

## 1 Introduction

Cobalt (Co) is a trace element, with average concentrations between 10 and 15 mg/kg in soil (Aubert and Pinta 1977). However, due to anthropogenic activities, such as industrial production of hard metals, grinding and mining (Agency for Toxicological Substances and Disease Registry [ATSDR] 2004; Gray and Eppinger 2012; Jiang et al. 2022), soil Co concentration in some areas has increased to a high level. For example, in the mining areas near the Idaho copper belt in the United States, the total Co concentration in the soil reached 940 mg/kg (Gray and Eppinger 2012), while it was 6253 mg/kg near the Katanga copper–cobalt hill in the Democratic Republic of Congo (Pourret et al. 2016).

Co is an essential element for prokaryotes (including cyanobacteria) and animals, but its essential role in plants has not been demonstrated, although a beneficial effect in symbiotic nitrogen fixation in some legumes was reported (Holm‐Hansen, Gerloff, and Skoog 1954; Ma et al. 2023). Co is a component of cobalamin, an essential vitamin (Vitamin B_12_). B_12_ acts as the co‐factor for two enzymes, that is, methylmalonyl‐CoA mutase and methionine synthase, in humans. Co is also a co‐factor of different enzymes and is a component of several proteins in prokaryotes and animals (Odaka and Kobayashi 2013). Non‐ruminants, including humans, have a requirement for cobalamin but cannot synthesize it by themselves (Hu et al. 2021; Ma et al. 2023). Only some bacteria, such as rumen microflora, can synthesize cobalamin (Ma et al. 2023). Therefore, in humans, direct intake of Co from plant foods cannot be utilized and is toxic in excess. Large amounts or chronic exposure to Co can cause various health problems, such as myocardial, neurological, endocrine and respiratory dysfunctions (Paley, Sobel, and Yalow 1958; Goldoni et al. 2004; Linna et al. 2004; Rizzetti et al. 2009). In plants, high Co concentration adversely affects their growth, photosynthesis and metabolic activity (Anjum et al. 2015; Lwalaba et al. 2019).

Diets such as vegetables and cereals are believed to be the main sources of non‐cobalamin Co exposure (Kim, Gibb, and Howe 2006; Leyssens et al. 2017). Therefore, it is a very important issue for human health to limit Co transfer from contaminated soil to the edible parts. However, it is poorly understood how Co is taken up from the soil and subsequently transported to the different organs of the plants. The major form of Co in soil solution is Co^2+^, so it has been proposed to be taken up by transporters for divalent cations, belonging to the Natural resistance‐associated macrophage protein (Nramp), the Zn‐regulated transporter, the Fe‐regulated transporter‐like protein (ZIP), heavy metal ATPase (HMA) and ferroportin (FPN) families. In fact, in *Arabidopsis*, Co^2+^ is taken up through a ferrous iron transporter, AtIRT1 (iron‐regulated transporter 1) (Vert et al. 2002). Once taken up, part of Co in the roots is sequestered into vacuoles by AtHMA3 and AtFPN2 (Morel et al. 2009; Morrissey et al. 2009), which are localized at the tonoplast, while the remaining part is loaded to the xylem by AtFPN1, which is a plasma membrane–localized Co transporter at the pericycle in *Arabidopsis* (Morrissey et al. 2009). Knockout of *AtIRT1* did not affect Co accumulation under Fe‐replete condition, but under Fe‐deficient condition, Co accumulation in the wild type (WT) increased due to increased expression of *AtIRT1*, whereas this increase was not observed in *irt1* mutant (Vert et al. 2002). Constitutive ectopic expression of *AtHMA3* leads to increased tolerance to high Co due to increased vacuolar sequestration of Co in the roots (Morel et al. 2009). On the other hand, knockout of *AtFPN1* decreased shoot Co accumulation, whereas knockout of *AtFPN2* increased shoot Co accumulation in *Arabidopsis* (Morrissey et al. 2009). A homologue of AtFPN1 in rice, OsFPN1 is also implicated in the root‐to‐shoot translocation of Co (Kan, Fujiwara, and Kamiya 2022). Recently, a member of rice ABC transporter family, ARG1 localized in the envelopes and thylakoid membrane of chloroplasts also transports Co^2+^ and is involved in Co homeostasis (Li et al. 2020).

Rice (*Oryza sativa*) is a staple food for nearly half of the world's population and is widely cultivated in many areas, including Co‐contaminated soil. Understanding how Co is taken up by the roots from soil, the first step of Co entering the plants, is of great importance for the control of Co accumulation in rice plants and the human body via the food chain. In the present study, we compared the Co accumulation between WT rice and several mutants defective in the uptake of Zn^2+^, Fe^2+^ or Mn^2+^. We found that Co uptake in rice is mediated by OsNramp5 for Mn^2+^, rather than IRT1 and ZIP9 for Fe^2+^ and Zn^2+^, respectively. Knockout of *OsNramp5* gene significantly decreased Co accumulation in rice plants and grains.

## 2 Materials and Methods

### 2.1 Plant Materials and Growth Conditions

Following mutants with their corresponding WT rice were used: two independent loss‐of‐function mutants of *OsNramp5* (*osnramp5‐1* and *osnramp5‐2*), *osirt1‐1* mutant and *oszip9‐1* mutant. *osnramp5‐1* (*lcd‐kmt2*) was produced by carbon ion‐beam irradiation from cultivar Koshihikari (WT1) (Ishikawa et al. 2012), while *osnramp5‐2* is a T‐DNA insertion mutant derived from cultivar ZH11 (WT2) (Sasaki et al. 2012). *osirt1‐1* was generated by the CRISPR/Cas9 technique as described below, while *oszip9‐1* was obtained from a previous study in Nipponbare (WT3) background (Huang, Sasaki, et al. 2020).

Seeds of WTs and mutants were soaked in water in the dark at 30°C for 2 days. The germinated seeds were then transferred onto a plastic net floating on a 0.5 mM CaCl_2_ solution in a 1.2‐L plastic pot. After 4–6 days of growth, the seedlings were transferred to a 3.5‐L plastic pot containing half‐strength Kimura B solution (Huang, Sasaki, et al. 2020). Freshly prepared FeSO_4_ was added at a final concentration of 2 μM. Plants were grown in a controlled greenhouse at 25°C–30°C with natural light. The nutrient solution was changed every 2 days. All experiments were performed with 3 to 10 biological replicates each.

### 2.2 Generation of *osirt1* Mutant

Twenty bases upstream of the PAM were selected as candidate target sequences (Supporting Information S1: Figure S1). The primers for the target sequence in the ORF region of *OsIRT1* are listed in Supporting Information S1: Table S1. The plant expression vector of Cas9 (pZDgRNA_Cas9ver.2_HPT) and single guide RNA expression vector (pU6gRNA) were used as described before (Che, Yamaji, and Ma 2021). The derived constructs were transformed into rice calluses (cv. Nipponbare, WT3) according to a previous study (Hiei et al. 1994).

To select the resultant mutants, we extracted genomic DNA from the leaves of transgenic rice plants, followed by PCR amplification using primer pairs flanking the designed target sites, as listed in Supporting Information S1: Table S1. The PCR products (~440 bp) were sequenced directly using the amplification primers mentioned above. A line with a 7 bp deletion (Supporting Information S1: Figure S1) was used for further analysis.

### 2.3 Physiological Characterization of Co Accumulation in Rice

To investigate Co accumulation in rice plants, we exposed seedlings (cv. Nipponbare, 23 days old) to half‐strength Kimura B solution containing 1, 2, 5 or 10 μM Co^2+^. The Co^2+^ used was prepared using cobalt chloride hexahydrate (CoCl_2_·6H_2_O) (Wako, Osaka, Japan). After 1‐day exposure, the roots were washed with pre‐cooled 5 mM CaCl_2_ solution three times and separated from the shoots. The samples harvested were subjected to digestion and Co determination by ICP‐MS as described below. Uptake and translocation rates were calculated as (content in plants/root dry weight) and (content in the shoot/whole content × 100), respectively.

### 2.4 Analysis of Co Accumulation in Different Rice Mutants

To investigate which transporters for divalent metals are involved in Co uptake, we compared Co accumulation between different mutants, including *osnramp5‐1*, *osirt1‐1*, *oszip9‐1* and their WTs. Seedlings (22 days old) were exposed to a nutrient solution containing 2 μM Co^2+^ and 1 μM germanium (Ge) as a control for 1 day. Plants were harvested as mentioned above and the samples were subjected to Co determination by ICP‐MS as described below.

### 2.5 Dose‐ and Time‐Dependent Accumulation of Co in *osnramp5* Mutants

To further characterize Co accumulation in *osnramp5* mutants, we performed a dose‐ and time‐dependent accumulation of Co using two independent mutants (*osnramp5‐1* and *osnramp5‐2*) and their WTs. For the dose–response experiment, seedlings (29 days old) were exposed to a nutrient solution containing 1, 2 and 5 μM Co^2+^ for 1 day. In a time‐dependent experiment, the seedlings (21, 19 and 15 days old) were exposed to 2 μM Co^2+^ for 1, 3 and 7 days, respectively, and harvested at the same day as mentioned above.

### 2.6 Xylem Sap and Root Cell Sap Collection

To further characterize Co uptake and accumulation, we compared Co concentration in the root cell sap and xylem sap between *osnramp5* mutants and WTs in a time‐dependent manner. After the seedlings (33 days old) of both mutants and WTs were exposed to a nutrient solution containing 2 μM Co^2+^ for 0.5, 1, 2, 4 and 8 h, the xylem sap and root cell sap were collected. To collect xylem sap, shoots were excised with a razor from 2 cm above the root–shoot junction, and the xylem sap was collected from the cut surface for 20 min by using a micropipette. Root cell sap was collected as described previously (Che, Yamaji, and Ma 2021). Xylem sap and root cell sap were subsequently diluted by 5% HNO_3_ (w/v) and subjected to Co determination as described below.

### 2.7 Effect of Knockout of *OsNramp5* on Co Accumulation in Rice Grain

To investigate the effect of knockout of *OsNramp5* on Co accumulation in rice grains, two independent *osnramp5* mutants and their WTs were cultivated in the soil pots amended with 5 mg Co^2+^/kg soil. The plants were cultivated until maturity. At harvest, the aboveground was separated into straw and grains after washing with deionized water three times. Agronomic traits such as plant height and dry weight of straw and grains were recorded. The concentration of Co and other elements in each organ was determined as described below.

### 2.8 Co Deposition Pattern Analysis by LA‐LCP‐MS

To observe Co distribution pattern in the roots, 5‐day‐old seedlings of two *osnramp5* mutants and their WTs were exposed to a 0.5 mM CaCl_2_ solution containing 2 μM Co^2+^ for 4 h. The roots (20 mm from the root apex) of each line were sampled and subjected to Co analysis with laser ablation (LA) device (NWR213; New Wave Research) and ICP‐MS (8900; Agilent Technologies) operated in helium mode. The sample preparation procedures and mapping method were the same as described by Yamaji and Ma (2019).

### 2.9 Effect of Co on Root Elongation of *OsNramp5* Knockout Lines

To evaluate the effect of Co on root elongation, 4‐day‐old seedlings of two independent *osnramp5* mutants and their WTs were exposed to a 0.5 mM CaCl_2_ solution containing 0, 1, 2, 5 or 10 μM Co^2+^ (pH 5.6) for 24 h. The root length was measured with a ruler before and after the treatment. Relative root elongation was calculated as (root elongation with Co treatment)/(root elongation without Co) × 100.

### 2.10 Co Transport Activity Assay in *Saccharomyces cerevisiae*

The transport activity of OsNramp5 was investigated by expressing this gene in yeast strain BY4741 according to a previous study (Yu et al. 2022). The transformed yeasts were grown on synthetic SD‐U medium containing 0.67% (w/v) yeast nitrogen base without amino acids (Difco), 0.19% (w/v) mixed amino acid without uracil, 0.003% (w/v) adenine hemisulphate, 2% (w/v) glucose and 2% (w/v) agar at pH 5.8 for selection. To test the transport activity, yeast expressing *OsNramp5* or empty vector was precultured in SD‐U liquid medium. The resuspended yeast cells were serially diluted at OD_600_ of 0.1, 0.01, 0.001 and 0.0001 with sterilized Milli‐Q water. A 5 μL of each dilution was spotted on SD‐U solid medium containing different concentrations of Co^2+^ in the presence of glucose. After incubation at 30°C for 48 h, the plates were photographed.

### 2.11 RNA Extraction and Gene Expression Analysis

To investigate the effect of Co exposure on *OsNramp5* expression, seedlings (cv. Nipponbare, 30 days old) were exposed to a nutrient solution with or without 2 μM Co^2+^ for 1 day. Roots were sampled and immediately frozen in liquid nitrogen. Total RNA was extracted from the roots using an RNeasy Plant Mini Kit (Qiagen) and then converted to cDNA with ReverTra Ace (Toyobo) according to the protocol supplied by the manufacturer. The expression level of *OsNramp5* was determined with KOD SYBR qPCR Mix (Toyobo) on a real‐time PCR machine CFX96 (Bio‐Rad). *HistoneH3* was used as an internal standard, and the relative gene expression was calculated by the ΔΔ*C*_t_ method. The primers used are listed in Supporting Information S1: Table S1.

### 2.12 Effect of Mn and Cd on Co Accumulation in Rice

To examine the effect of Mn supply on Co accumulation, seedlings (21 days old) of WT1 were exposed to a nutrient solution containing 2 μM Co^2+^ in the presence of 0.5, 5 or 50 μM Mn^2+^. To investigate the effect of Cd supply on Co accumulation, 23‐day‐old seedlings of WT1 were exposed to a nutrient solution containing 2 μM Co^2+^ in the presence of 0, 0.5, 1 or 2 μM Cd^2+^. After 1‐day exposure, the shoots and roots were separately harvested after the roots were washed with pre‐cooled 5 mM CaCl_2_ solution three times and subjected to Co determination as described below.

### 2.13 Co and Other Elements Determination

Plant samples harvested were dried at 70°C for at least 2 days, and then digested by 61% HNO_3_ (w/v). The concentration of Co and other elements in the digestion solution, root cell sap and xylem sap was determined with ICP‐MS (7700X and 8900; Agilent Technologies).

### 2.14 Statistical Analysis

Statistical comparison was performed by Student's *t*‐test or ANOVA, followed by Duncan's test. The significance of differences was defined as: *p < 0.05, **p < 0.01 or different letters (*p* < 0.05).

## 3 Results

### 3.1 Physiological Characterization of Co Accumulation in Rice

**Figure 1:**
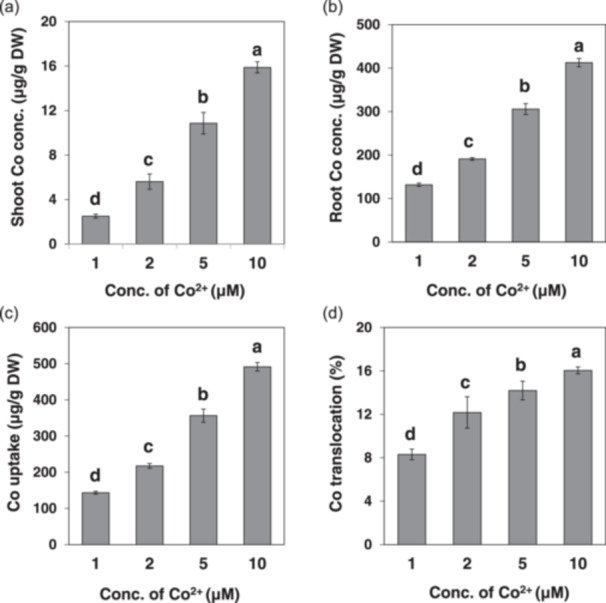
Physiological characterization of Co accumulation in rice. (a–d) Dose‐dependent accumulation of Co in the shoots (a), roots (b), uptake (c) and root‐to‐shoot translocation (d). Seedlings (23 days old, cv. Nipponbare) were exposed to a solution containing 1, 2, 5 or 10 μM Co^2+^ for 1 day. The concentration of Co in shoots and roots was determined by ICP‐MS after digestion. Uptake (c) was calculated by Co content in plants/root dry weight. The ratio of root‐to‐shoot translocation (d) was calculated by Co content in shoots/total Co content × 100. Data are expressed as means ± SD of three biological replicates. Statistical comparison was performed by ANOVA, followed by Duncan's test. Bars with different letters indicate significant differences (*p* < 0.05). Conc., concentration; DW, dry weight.

Since Co^2+^ is the major form in soil (Wendling, Kirby, and McLaughlin 2008), we investigated Co accumulation in the roots and shoots at different Co^2+^ concentrations ranging from 1 to 10 µM. The Co concentration in both roots and shoots increased with increasing Co^2+^ concentrations in the solution (Figure 1). The Co concentration in the roots was much higher than that in the shoots (Figure 1), resulting in low root‐to‐shoot translocation (8%–16%).

### 3.2 Searching for Co Uptake Transporter

**Figure 2:**
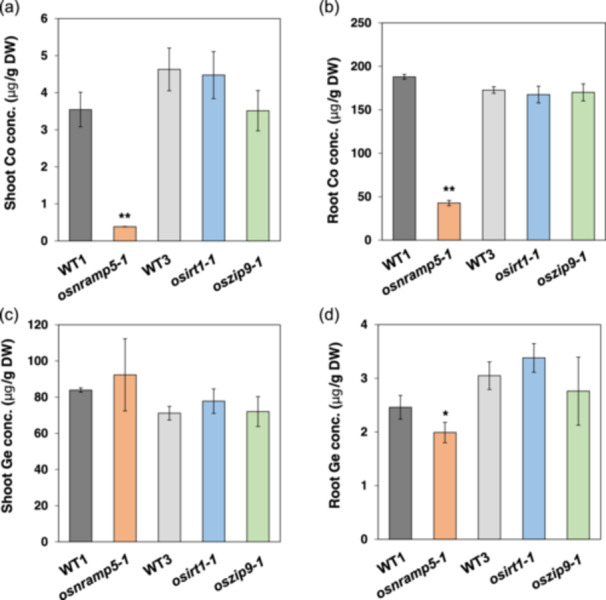
Effect of knockout of *OsNramp5*, *OsIRT1* and *OsZIP9* on Co accumulation in rice. Twenty‐two‐day‐old seedlings of *osnramp5‐1* mutant and its WT (Koshihikari [WT1]), *osirt1‐1* mutant and *oszip9‐1* mutant as well as their WT (Nipponbare [WT3]) were exposed to a nutrient solution containing 2 μM Co^2+^ and 1 μM Ge for 1 day. The concentration of Co (a and b) and Ge (c and d) in the roots and shoots was determined by ICP‐MS. Data are expressed as means ± SD of three biological replicates. Significant differences between *osnramp5‐1*, *osirt1‐1* and *oszip9‐1* mutants and their corresponding WTs are marked with *p < 0.05 and **p < 0.01, by Student's *t*‐test. Conc., concentration; DW, dry weight.

So far, transporter specific for Co^2+^ uptake has not been identified in rice, but it was reported that Co^2+^ shares the same transporters for other divalent metals such as Mn^2+^, Fe^2+^, Cd^2+^ and Zn^2+^ in *Arabidopsis* (Vert et al. 2002). To search for transporter involved in Co uptake in rice, we first compared Co accumulation between WT rice and several mutants defective in divalent metal uptake including *osnramp5*, *osirt1* and *oszip9*. OsNramp5 is a transporter involved in Mn/Cd uptake (Sasaki et al. 2012), while OsZIP9 is a Zn transporter for Zn uptake (Huang, Sasaki, et al. 2020). OsIRT1 shows transport activity for Fe^2+^ in yeast, although its role in rice has not been investigated (Ishimaru et al. 2006). When these mutants with their WTs were exposed to a nutrient solution containing 2 μM Co^2+^ for 1 day, only *osnramp5* mutant showed lower Co accumulation in both roots and shoots compared with the WT, whereas the other two mutants (*osirt1‐1* and *oszip9‐1*) showed similar Co accumulation in the roots and shoots as their WT (Figure 2a,b). To make sure that these differences in Co accumulation result from the defect of OsNramp5, we also compared the accumulation of Ge, an analogue of Si as a control. The results showed that there was no difference in Ge accumulation in the shoots between all mutants and their WTs, although *osnramp5‐1* mutant showed lower Ge concentration in the roots compared with other mutants probably due to inhibited root growth (Figure 2c,d).

### 3.3 Characterization of Co Accumulation in *osnramp5* Mutants

To confirm the role of OsNramp5 in Co^2+^ uptake, we performed a time‐ and dose‐dependent experiments using two independent *osnramp5* mutants with different genetic backgrounds.

**Figure 3:**
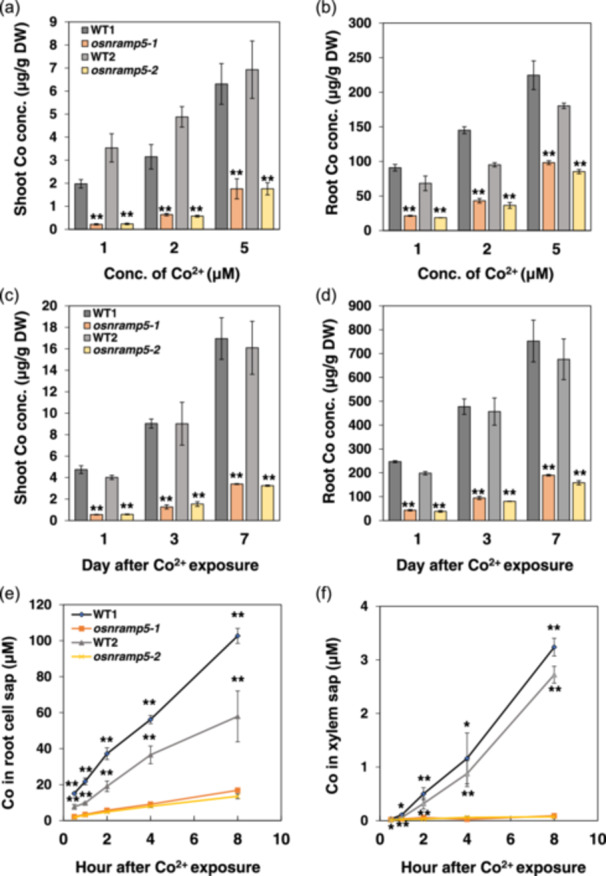
Effect of knockout of *OsNramp5* on Co accumulation in rice. (a and b) Dose‐dependent Co concentration in the shoots (a) and roots (b). Twenty‐nine‐day‐old seedlings of *osnramp5* mutants (*osnramp5‐1* and *osnramp5‐2*) and their WTs (Koshihikari [WT1] and ZH11 [WT2]) were exposed to 1, 2 or 5 μM Co^2+^ for 1 day. (c and d) Time‐dependent Co concentration in the shoots (c) and roots (d). Seedlings of *osnramp5* mutants and their WTs were exposed to 2 μM Co^2+^ for 1, 3 and 7 days, respectively. (e and f) Co in the root cell sap and xylem sap. Thirty‐three‐day‐old seedlings of *osnramp5* mutants and their WTs were exposed to 2 μM Co^2+^ for 0.5, 1, 2, 4 and 8 h, respectively. The concentrations of Co in the roots and shoots, root cell sap and xylem sap were determined by ICP‐MS. Data are expressed as means ± SD of three biological replicates. Significant differences between *osnramp5* mutants and their corresponding WTs are marked with *p < 0.05 and **p < 0.01, by Student's *t*‐test. Conc., concentration; DW, dry weight. [Color figure can be viewed at wileyonlinelibrary.com]

In a dose‐dependent experiment, the Co concentration in both roots and shoots was much lower in the mutants than in their WTs (Figure 3a,b), being 23%–47% and 7%–28% of the WTs in the roots and shoots, respectively. A time‐course experiment also showed that knockout of *OsNramp5* significantly decreased Co accumulation in the roots and shoots (Figure 3c,d).

Furthermore, we compared the Co concentration in the root cell sap between mutants and their WTs after a short‐term (up to 8 h) exposure to Co^2+^. The Co concentration in the root cell sap linearly increased with exposure time in the WTs, but this increase was much less in the mutants (Figure 3e). At 8 h after the exposure, the Co concentrations in the root cell sap were three to five times higher in the WT than in the mutants (Figure 3e).

We also compared Co concentration in the xylem sap. The Co concentration in the xylem sap was much lower compared with the root cell sap, but the WTs showed much higher Co in the xylem sap than the mutants at all time points (Figure 3f). At 8 h after Co exposure, the Co concentrations in the xylem sap were 35–36 times higher in the WTs than that in the mutants (Figure 3f).

### 3.4 Deposition Pattern of Co in Roots

**Figure 4:**
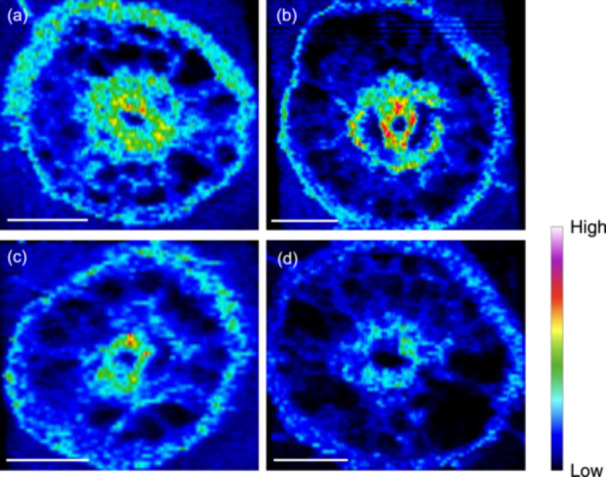
Co deposition in rice root. (a–d) Deposition pattern of Co in roots of both WTs (WT1 [a] and WT2 [c]) and mutants (*osnramp5‐1* [b] and *osnramp5‐2* [d]). Five‐day‐old seedlings were exposed to a solution containing 2 μM Co^2+^ for 4 h. Roots (20 mm from the root apex) of each line were sampled for Co mapping by LA‐ICP‐MS. Scale bars = 100 μm. [Color figure can be viewed at wileyonlinelibrary.com]

With the help of LA‐ICP‐MS, we mapped the deposition of Co in the roots of the WT and mutants. Deposition of Co in the roots was observed in most tissues with stronger signal at the exodermis, endodermis and stele region (Figure 4a,c). There was no difference in the deposition pattern between WT and mutants; however, a much weaker signal of Co was detected in the roots of the *osnramp5* mutants compared with those of WTs (Figure 4).

### 3.5 Co Toxicity in *osnramp5* Mutants

**Figure 5:**
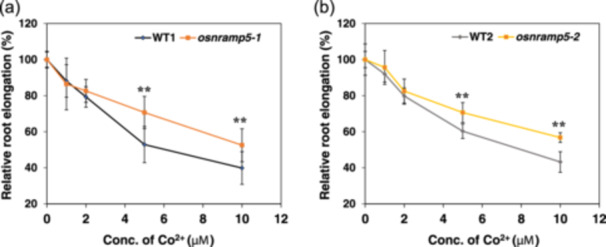
Effect of Co on root elongation of *osnramp5* mutants and their wild‐type (WT) rice. (a and b) Relative root elongation. Seedlings (4 days old) of two *osnramp5* mutants (*osnramp5‐1* [a] and *osnramp5‐2* [b]) and their WTs (WT1 and WT2) were exposed to a solution (pH 5.6) containing 0, 1, 2, 5 or 10 μM Co^2+^ for 24 h. The root length was measured before and after the treatment. Root elongation relative to no Co treatment is shown. Data are expressed as means ± SD of 10 biological replicates. Significant differences between *osnramp5* mutants and their corresponding WTs are marked with **p < 0.01, by Student's *t*‐test. Conc., concentration. [Color figure can be viewed at wileyonlinelibrary.com]

To test whether decreased Co deposition in the mutant roots is associated with Co toxicity tolerance, we compared root elongation during 24 h at different Co^2+^ concentrations. The root elongation of both WTs and mutants was inhibited with increasing Co^2+^ concentrations supplied (Figure 5); however, less inhibition was found in the mutants, especially at higher Co concentrations (5–10 μM). These results are consistent with Co deposition and accumulation in the roots (Figures 3 and 4).

### 3.6 Transport Activity of OsNramp5 for Co^2+^ in Yeast

**Figure 6:**
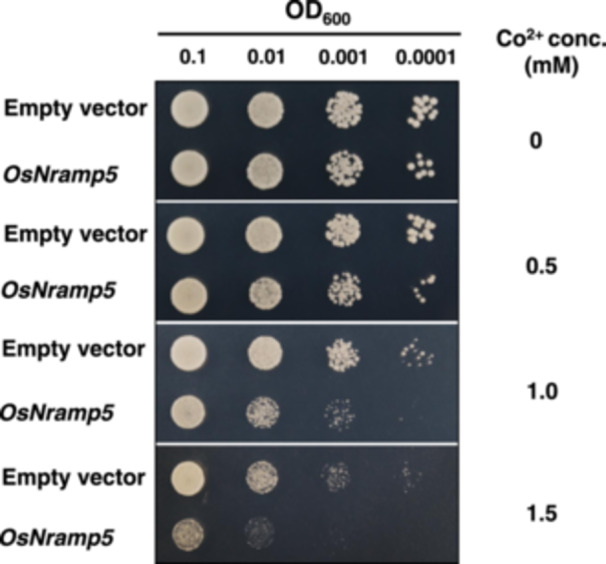
Transport activity of OsNramp5 for Co^2+^ in yeast cells. Different dilution of yeast strain expressing an empty vector or *OsNramp5* was spotted on a SD‐U solid medium containing different concentrations of Co^2+^. After 48‐h growth, the plates were photographed. Conc., concentration. [Color figure can be viewed at wileyonlinelibrary.com]

To test whether OsNramp5 can transport Co^2+^, we expressed *OsNramp5* in yeast cells (BY4741). In the absence of Co^2+^, no growth difference was found between yeast expressing *OsNramp5* and empty vector (Figure 6). However, in the presence of Co^2+^, the growth of yeast expressing *OsNramp5* was inhibited more compared with the empty vector, especially at higher concentrations (> 1 mM). These results indicate that OsNramp5 has transport activity for Co^2+^.

### 3.7 Effect of Co on *OsNramp5* Expression

We examined whether the expression of *OsNramp5* in the roots is altered by the presence of Co^2+^ or not by real‐time RT‐PCR. The result showed that the expression level of *OsNramp5* was similar in the roots exposed to Co or not, indicating its constitutive expression in the roots (Supporting Information S1: Figure S2).

### 3.8 Interaction Between Co and Mn or Cd

**Figure 7:**
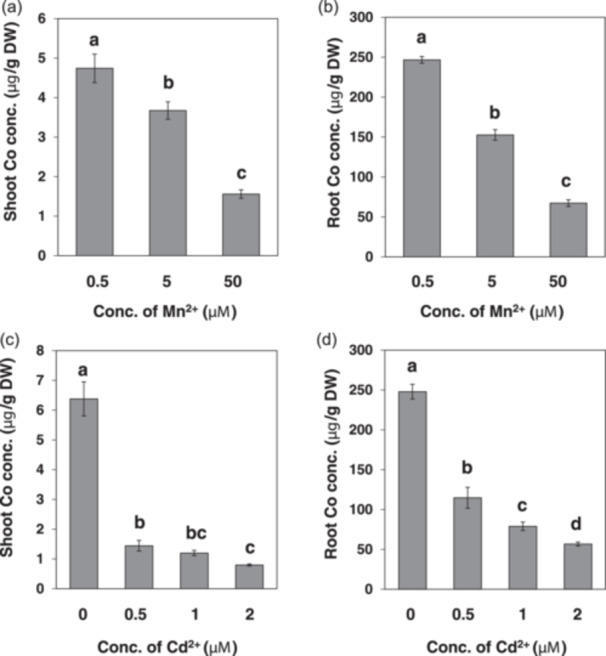
Effect of Mn and Cd on Co accumulation in rice. (a and b) Effect of Mn on Co accumulation. Twenty‐one‐day‐old seedlings (cv. Koshihikari) were exposed to a nutrient solution containing 2 μM Co^2+^ in the presence of 0.5, 5 or 50 μM Mn^2+^ for 1 day. (c and d) Effect of Cd on Co accumulation. Twenty‐three‐day‐old seedlings (Koshihikari) were exposed to a nutrient solution containing 2 μM Co^2+^ in the presence of 0, 0.5, 1 or 2 μM Cd^2+^ for 1 day. The concentration of Co was determined by ICP‐MS. Data are expressed as means ± SD of three biological replicates. Statistical comparison was performed by ANOVA, followed by Duncan's test. Bars with different letters indicate significant differences (*p* < 0.05). Conc., concentration; DW, dry weight.

Since OsNramp5 also mediates the uptake of Mn^2+^ and Cd^2+^, we investigated the interaction between Co^2+^ and Mn^2+^ or Cd^2+^ in terms of Co accumulation in the roots and shoots. Co concentration in both the roots and shoots was gradually decreased with increasing external Mn^2+^ concentrations from 0.5 to 50 μM (Figure 7a,b). At 50 μM Mn^2+^, the Co concentration in the roots and shoots was only 27% and 33%, respectively, of that at 0.5 μM Mn^2+^ (Figure 7a,b).

The Co concentration in the roots and shoots was also decreased with increasing Cd^2+^ concentration in the external solution (Figure 7c,d). Furthermore, it seems that Cd^2+^ was more effective at reducing Co accumulation in the roots and shoots compared with Mn because Cd^2+^ decreased more Co than Mn^2+^ at the same concentration (0.5 μM) (Figure 7). These results indicate the competition between Co^2+^ and Mn^2+^/Cd^2+^ occurred for the uptake.

### 3.9 Co Accumulation in Rice Grain of Mutants

**Figure 8:**
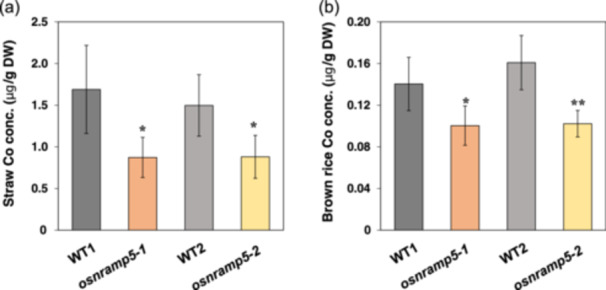
Effect of knockout of *OsNramp5* on Co concentration in straw and brown rice. (a and b) Co concentration in straw (a) and brown rice (b) of *osnramp5* mutants (*osnramp5‐1* and *osnramp5‐2*) and their WTs (Koshihikari [WT1] and ZH11 [WT2]). The plants were grown in Co‐contaminated soil until maturity. The Co concentration was determined by ICP‐MS. Data are expressed as means ± SD of four biological replicates. Significant differences between *osnramp5* mutants and their corresponding WTs are marked with *p < 0.05 and **p < 0.01, by Student's *t*‐test. Conc., concentration; DW, dry weight. [Color figure can be viewed at wileyonlinelibrary.com]

To investigate the effect of knockout of *OsNramp5* on the Co accumulation in rice grain, we grew both two independent mutants and their WTs in the soil pots amended with 5 mg Co^2+^/kg soil until maturity. Element analysis showed that *osnramp5* mutants accumulated much less Co in the straw than the WTs, being only 52%–59% of the WTs (Figure 8a). In the grain, the Co concentration was decreased by 28%–36% due to knockout of *OsNramp5* compared with the WTs (Figure 8b). Similar to previous studies (Sasaki et al. 2012; Chang et al. 2022), the mutants showed lower plant height, less biomass of straw and grain yield (Supporting Information S1: Figure S3) and lower accumulation of Mn and Cd in the straw and grain (Supporting Information S1: Figure S4).

## 4 Discussion

With increasing health risks due to Co contamination in soil, it is important to reduce Co accumulation in edible parts, especially in staple foods such as rice. Some approaches have been reported to reduce Co accumulation in plants. For example, the application of manure and calcium oxide decreased Co accumulation in the shoots of spring barley by stabilizing Co in the soil (Kosiorek and Wyszkowski 2020). Engineered biochar application reduced Co concentration in wheat shoots by increasing pH and cation exchange capacity (CEC) to decrease Co bioavailability in soil (Mohamed et al. 2021). However, these approaches are generally time‐consuming and not cost‐effective (Sharma et al. 2018). One of the best and sustainable ways to reduce Co accumulation is to breed cultivars with low Co accumulation. For this purpose, it is a prerequisite to understand the molecular mechanisms on how Co is taken up and how Co is finally accumulated into the edible parts. There are several steps for transporting Co from soil to edible parts, including uptake, vacuolar sequestration in the root cells, root‐to‐shoot translocation, distribution and redistribution to the edible parts (Huang, Wang, et al. 2020). In the present study, we found that Co uptake in rice, the first step entering plants, is mediated by OsNramp5.

OsNramp5 belongs to the Nramp family, which is known to transport divalent cations (Colangelo and Guerinot 2006). In rice, there are seven members of Nramp family, which show different transport substrates. For example, OsNramp3 transports only Mn^2+^ (Yamaji et al. 2013), while OsNramp1 transports both Mn^2+^ and Cd^2+^ (Chang et al. 2020). Interestingly, OsNramp4 (Nrat1) transports Al^3+^ (Xia et al. 2010). OsNramp5 was initially reported as a Mn/Cd transporter in rice (Sasaki et al. 2012), but later it was also found to be able to transport Pb (Chang et al. 2022). OsNramp5 is a plasma membrane–localized protein and is polarly localized at the distal side of both exodermis and endodermis in the roots (Sasaki et al. 2012). Recently, it was found that *OsNramp5* is also expressed in the leaf sheath and is involved in the local distribution of Mn between the leaf sheath and blade (Huang et al. 2024). In the present study, we found that OsNramp5 also transports Co^2+^ when expressed in yeast (Figure 6). Knockout of this gene almost lost the ability to take up Co^2+^ (Figures 3b,d,e and 4), and reduced subsequent accumulation in the shoots (Figure 3a,c,f). These results indicate that OsNramp5 is a major transporter for Co uptake in rice, although there may be other specific, unknown transporter for Co with less contribution. In *Arabidopsis*, Co uptake is medicated by IRT1, a transporter for divalent metals including Fe, Mn and Zn (Vert et al. 2002). These findings suggest that the transporter involved in Co uptake differs with plant species. It will be interesting to identify more Co transporters in other plant species in the future.

Due to the distinct structure of rice roots, both influx and efflux transporters are required for an efficient uptake of mineral elements. For example, Mn uptake requires both OsNramp5 and OsMTP9, which are, respectively, polarly localized at the distal and proximal side of both exodermis and endodermis in the roots (Sasaki et al. 2012; Ueno et al. 2015). Although OsNramp5 is responsible for transporting Co from soil to the root cells, the efflux transporter for Co remains unknown. OsMTP9 as a Mn efflux transporter seems not to be involved in Co efflux because knockout of this gene did not affect Co uptake (Ueno et al. 2015). In *Arabidopsis*, AtFPN1 localized at the plasma membrane of stele cells was reported to be involved in xylem loading, while its homologue in rice OsFPN1 is localized at the Golgi, although knockout of this gene also resulted in decreased root‐to‐shoot translocation of Co (Morrissey et al. 2009; Kan, Fujiwara, and Kamiya 2022). Because Co has not been recognized as an essential element for plant growth, although it exerts beneficial effects at low concentrations, especially in leguminous plants (Pilon‐Smits et al. 2009), plants may not have specific efflux transporters for Co and may share transporters for essential metals with similar chemical properties. However, given the fact that the Co concentration in the xylem sap was only 3%–5% of that in the root cell sap (Figure 3e,f), it seems that there is no specific efflux transporter for efficiently transporting Co toward the stele, although further works are required. This is supported by low root‐to‐shoot translocation of Co (Figure 1d) and heavy deposition of Co in the roots, including the outer layer, inner layer and stele region (Figure 4a,c).

The high Co in root cell sap also suggests that most Co taken up is sequestrated into the vacuoles in the root cells. In *Arabidopsis*, AtHMA3 seems to be involved in Co sequestration to the vacuoles because overexpression of this gene resulted in increased tolerance to high Co, although knockout of this gene did not show the difference in Co toxicity tolerance (Morel et al. 2009). In rice, a homologue of AtHMA3, OsHMA3, is involved in the sequestration of Cd and Zn into vacuoles (Ueno et al. 2010; Cai et al. 2019), but it is unclear whether it also functions in vacuolar Co sequestration.

Knockout of *OsNramp5* significantly decreased Co accumulation in rice grain (Figure 8b), offering a strategy for limiting Co transport to rice grain. However, there is also a risk of negative impact on the grain yield due to decreased Mn uptake (Supporting Information S1: Figures S3 and S4), although controversial results on yield penalty were reported (Ishikawa et al. 2012; Sasaki et al. 2012; Tang et al. 2017). Manipulation of transport selectivity may solve this problem in the future. Identification of key amino acid residues for the selectivity of Co and Mn will be important for breeding rice cultivars with low Co accumulation. Recently, a weak mutant allele of OsNramp5 decreasing transport activity for both Mn and Cd was identified (Kuramata et al. 2022). On the other hand, gene duplication of *OsNramp5* was reported to decrease the accumulation of Cd, but not Mn, in rice grain without growth and yield penalties (Yu et al. 2022). It will be interesting to test whether these alleles also decrease Co accumulation in the future. In addition, since Mn competes with Co for OsNramp5 during uptake (Figure 7a,b), supplying Mn to the soil may also decrease Co accumulation in rice grains.

In conclusion, we found that Co^2+^ uptake in rice is mediated by OsNramp5. Knockout out of this gene significantly decreases Co accumulation in rice plants and grains. Manipulation of selectivity of this transporter will provide a sustainable way for reducing Co accumulation in rice grain through applying this knowledge to breeding.

## Conflicts of Interest

The authors declare no conflicts of interest.

## Supporting information

Supporting information.

## Data Availability

The data that support the findings of this study are available on request from the corresponding author. The data are not publicly available due to privacy or ethical restrictions.
