# Supplementary material for: Uptake and Accumulation of Cobalt Is Mediated by OsNramp5 in Rice
Source: Plant Cell Environ. 2024 Sep 2;48(1):3–14. doi: 10.1111/pce.15130 (PMC11615428; doi:10.1111/pce.15130)
Supplement: Supplementary file 1 — Supporting information. [file PCE-48-3-s001.pdf]

Supporting Information

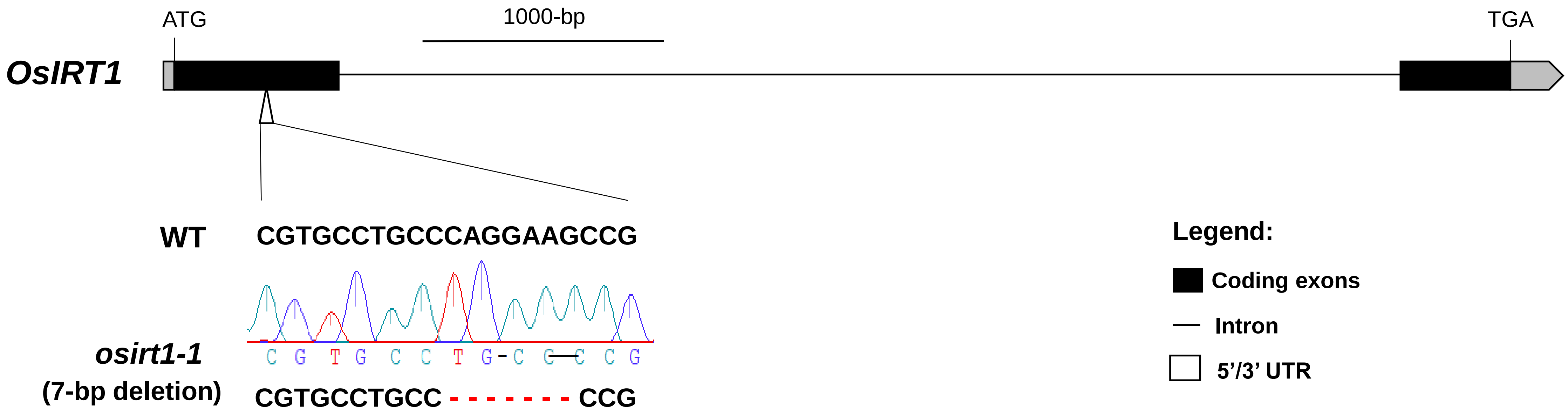

Figure S1. Gene structure of *OsIRT1* and its mutated sequence of CRISPR/Cas9 mutant.

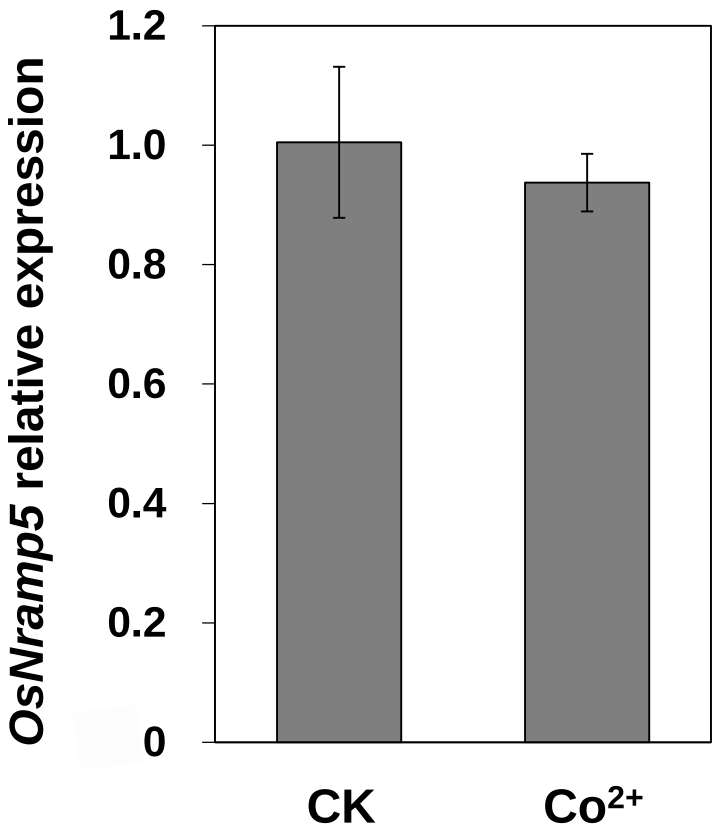

Figure S2. Effect of Co exposure on the expression of *OsNramp5* in rice roots. 30-d-old seedlings of Nipponbare were exposed to nutrient solution with or without 2 μM Co^{2+} for 1 d. The roots were sampled for RNA extraction and gene expression level of *OsNramp5* was determined. Data are means ± SD of 4 biological replicates.

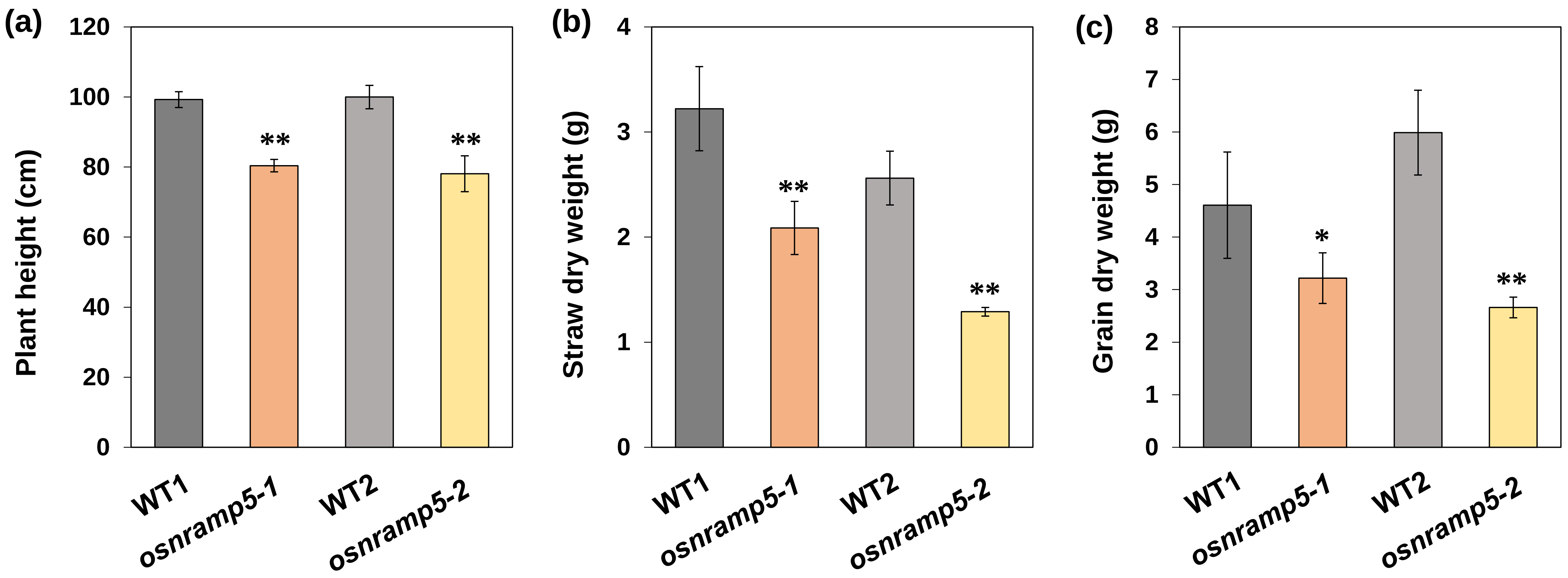

Figure S3. Agronomic traits of *osnramp5* mutants (*osnramp5-1* and *osnramp5-2*) and their WTs (Koshihikari (WT1) and ZH11 (WT2)) grown in Co-contaminated soil. Plant height (a) was recorded after plants were harvested from soil pots. Dry weights of straw (b), and grain (c) were measured after samples were dried up. Data are means ± SD of 4 biological replicates. Significant differences between *osnramp5* mutants and their corresponding WTs are marked with *$P < 0.05$ and **$P < 0.01$, by Student's *t*-test.

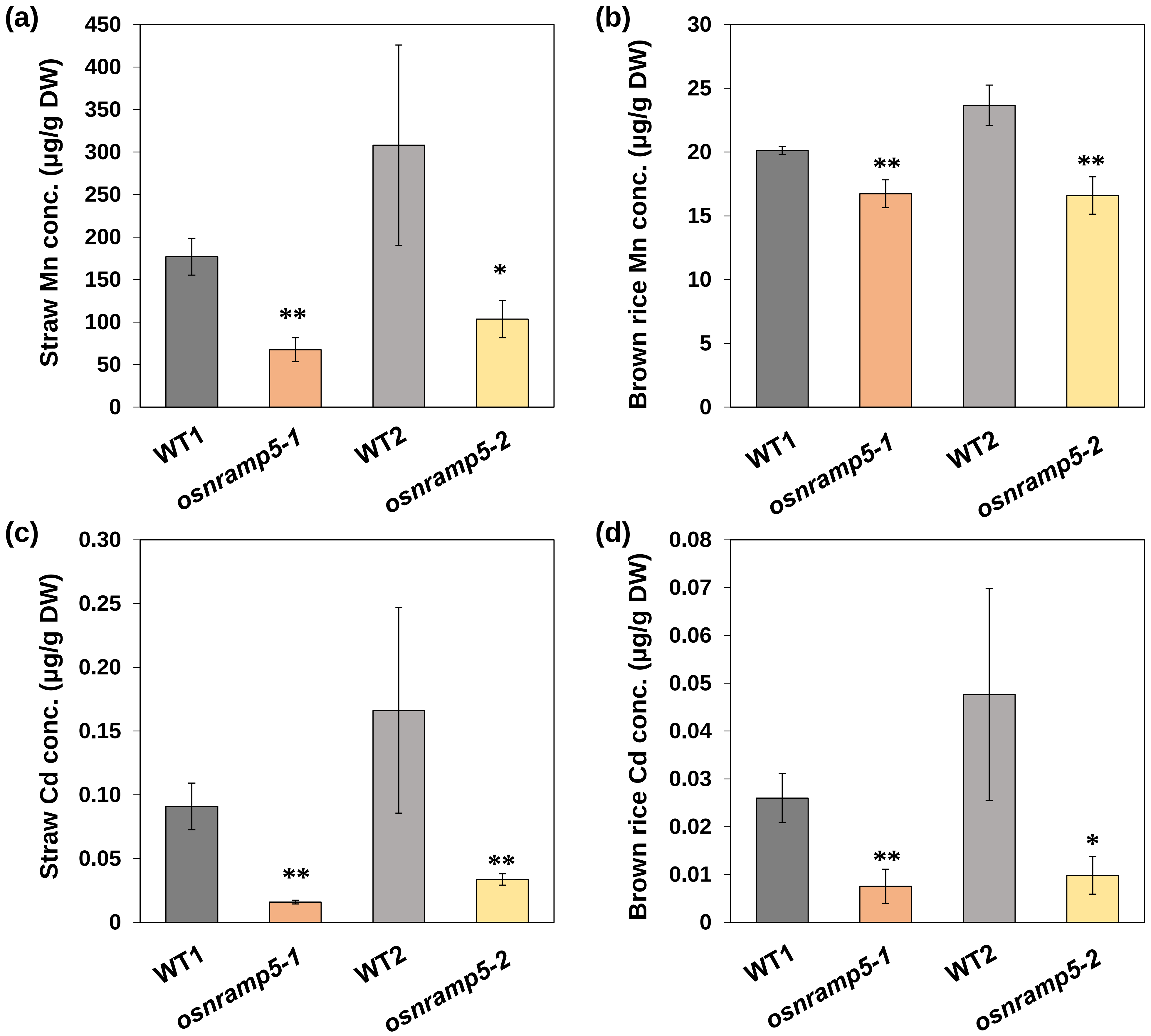

Figure S4. Effect of knockout of *OsNramp5* on Mn (a-b) and Cd (c-d) concentration in straw (a and c) and brown rice (b and d) grown in Co-contaminated soil. Mn and Cd in straw and brown rice of *osnramp5* mutants and their WTs (Koshihikari (WT1) and ZH11 (WT2)) were determined by ICP-MS. Data are means ± SD of 4 biological replicates. Significant differences between *osnramp5* mutants and their corresponding WTs are marked with *$P < 0.05$ and **$P < 0.01$, by Student's *t*-test. Conc., concentration; DW, dry weight.

Table S1. Primers used in this study

**Primer name**	**Forward (5'-3')**	**Reverse (5'-3')**	**Purpose**
OsIRT1-crispr	gttgCGTGCCTGCCCAGGAAGCCG	aaacCGGCTTCCTGGGCAGGCACG	CRISPR/CAS9 system
OsIRT1-crispr-check	TCTTCGCCGTCGTCAAGG	GTGTGTGATGTGAGGTTACAGCTTT	Homozygous mutant identification
OsNramp5 (qRT)	CAGCAGCAGTAAGAGCAAGATG	GTGCTCAGGAAGTACATGTTGAT	Gene expression
HistoneH3	GGTCAACTTGTTGATTCCCCTCT	AACCGCAAAATCCAAAGAACG	
